# Supplementary material for: Mechanistic Studies and a Retrospective Cohort Study: The Interaction between PPAR Agonists and Immunomodulatory Agents in Multiple Myeloma
Source: Cancers (Basel). 2022 Oct 27;14(21):5272. doi: 10.3390/cancers14215272 (PMC9657746; doi:10.3390/cancers14215272)
Supplement: Supplementary file 1 [file cancers-14-05272-s001.zip › cancers-1952052-supplementary.pdf]

Mechanistic studies and a retrospective cohort study: the interaction between PPAR agonists and immunomodulatory agents in multiple myeloma

Jian Wu[1], Emily Chu[1], Barry Paul[1], Yubin Kang[1,*]

[1] Division of Hematologic Malignancies and Cellular Therapy, Department of Medicine, Duke University Medical Center, Durham, North Carolina, USA.

***Corresponding author**

Yubin Kang

Email: yubin.kang@duke.edu

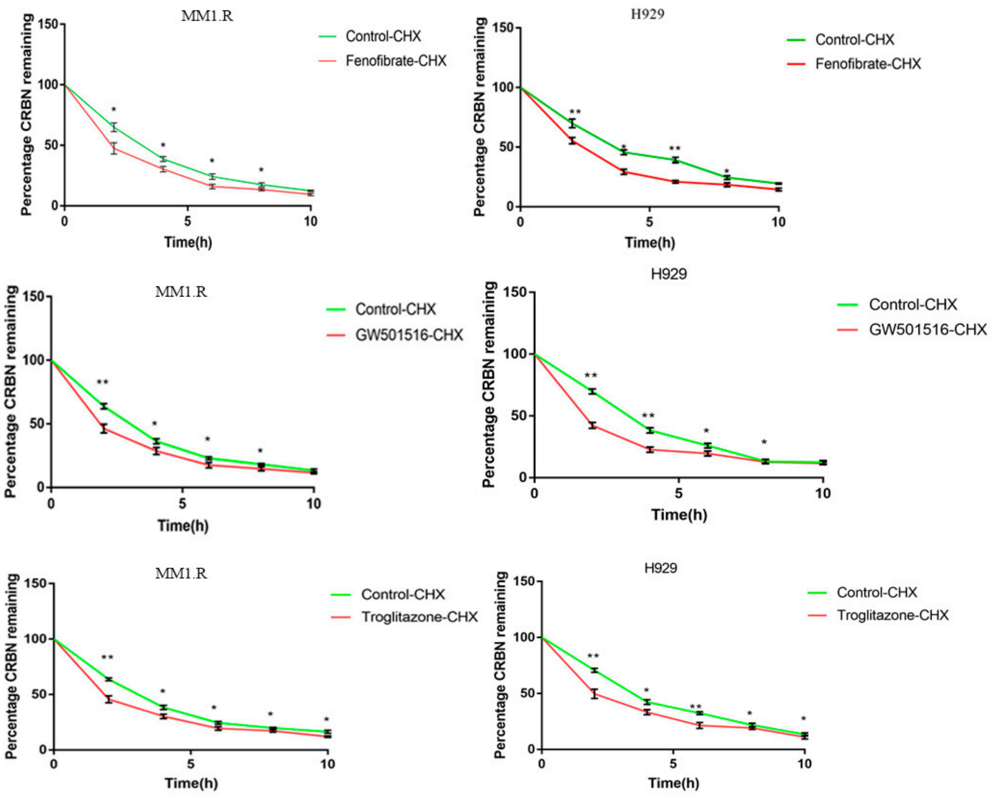

Figure S1. PPARs agonist increase CRBN protein degradation. The immunoblots were quantified by densitometric analysis. The intensities of the CRBN were normalized to the intensities for GAPDH. All values are shown as means SD of three independent experiments. *: $p<0.05$, **: $p<0.01$.

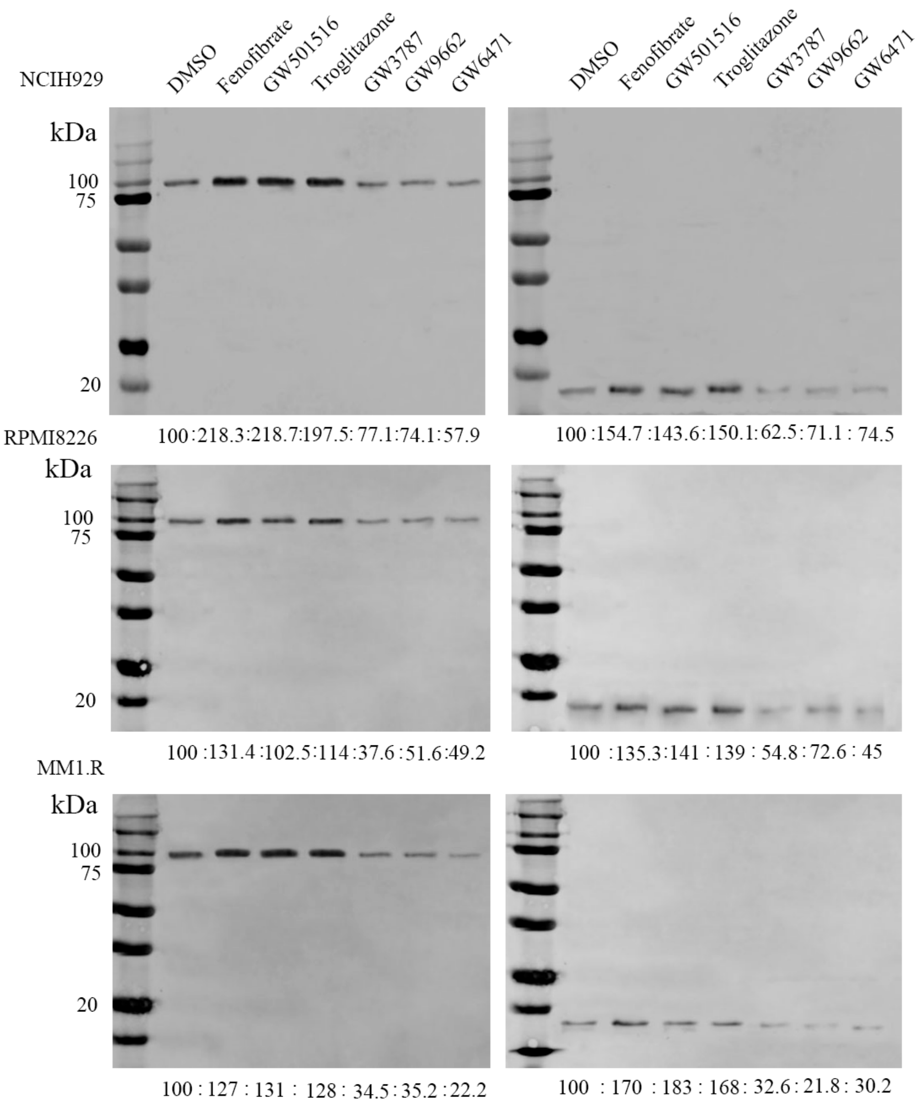

Figure S2. Uncropped blots and intensity ratio of Figure 2C.

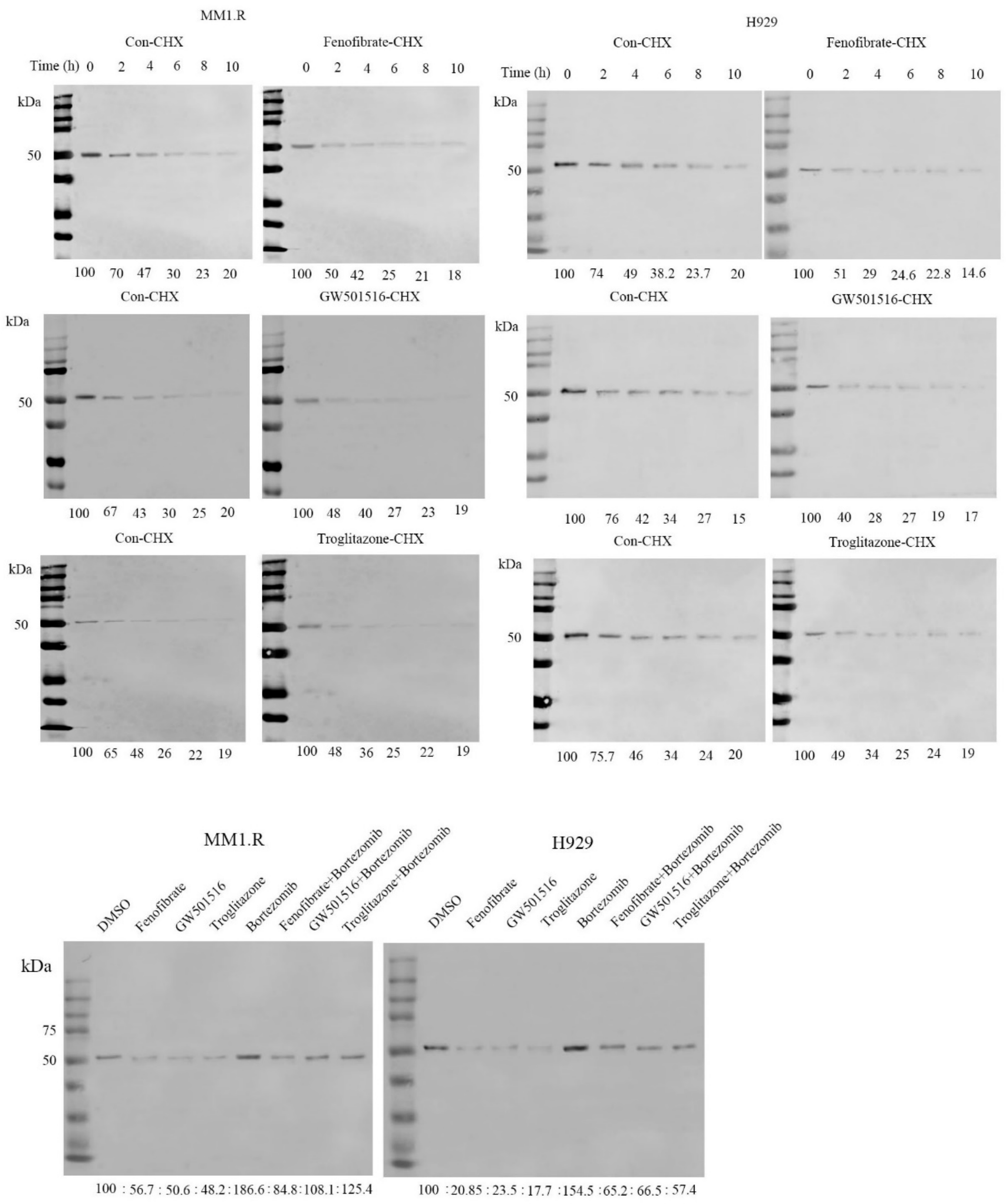

Figure S3. Uncropped blots and intensity ratio of Figure 3.

Supplementary Table S1. Clinical characteristics of patients treated with IMiD and with/without PPAR agonist.

	IMiD (n=114)	IMiD/PPAR agonist (n=82)	p value
**Mean Age**	64.5	64.7	0.87
**Gender, n (%)**			
Female	46 (40.4%)	22 (26.8%)	0.541921
Male	68 (59.6%)	60 (73%)	
**Race, n (%)**			
Caucasian	62 (54.4%)	58 (70.7%)	0.652194
African American	41 (36%)	21 (25.6%)	
Other	11 (9.6%)	3 (3.7%)	
**BMI, n (%)**			
18.5-24.9	31 (23.7%)	18 (21.9%)	0.238717
25-29.9	38 (33.3%)	24 (29.3%)	
>30	45 (35.1%)	40 (48.8%)	
**Subtype, n (%)**			
IgA-K	10 (8.7%)	9 (11%)	0.522319
IgA-L	10 (8.7%)	9 (11%)	
IgG-K	43 (37.7%)	27 (33%)	
IgG-L	23 (20.2%)	20 (24.4%)	
Lambda LC	5 (4.4%)	4 (4.9%)	
Kappa LC	2 (1.7%)	1 (1.2%)	
Unknown/Biclonal	21 (18.4%)	12 (14.6%)	
**ISS stage, n (%)**			
1	24 (21.1%)	10 (12.2%)	0.493987
2	31 (27.2%)	11 (13.4%)	
3	17 (15%)	12 (14.6%)	
Unknown	44 (38.6%)	50 (61%)	
**Cytogenetics, n (%)**			
Standard-risk	61 (53.5%)	36 (43.9%)	0.571215
High-risk	15 (13.2%)	21 (25.6%)	
intermediate	8 (7.0%)	5 (6.1%)	
unknown	30 (26.3%)	20 (24.4%)	
**Autologous HSCT, n (%)**			

Autologous HSCT	62 (54.4%)	45 (54.9%)	0.0913
**Overall Survival (median, months)**	114	70.2	0.0416
**Progression free survival (median, months)**	37.9	21.3	0.0374

Supplementary Table S2. Overall response rate in myeloma patients treated with IMiD alone or with concurrent PPAR agonists.

	IMiD (%) (n=114)	IMiD/PPAR agonist (%) (n=82)	p value
**Response**			
CR	28 (24.6)	9 (11.0)	
VGPR	40 (35.1)	22 (26.82)	
PR	35 (30.7)	22 (26.82)	
SD	2 (1.8)	6 (7.3)	
PD	5 (4.4)	17 (20.7)	
Unknown	5 (4.4)	6 (7.3)	
**Overall response rate** (CR+VGPR+PR)	90.4	64.6	<0.0001

Supplemental Table S3. Multivariable cox regression analysis

	P value	Hazard ratio (HR)	95.0% CI for HR	
			Lower	Upper
Age	0.033	0.501	0.265	0.946
Response	0.143	1.156	0.952	1.403
Cytogenetic	0.232	1.123	0.929	1.357
BMI	0.043	1.382	1.010	1.893
ISS Stage	0.078	1.224	0.978	1.532
M protein	0.339	1.070	0.932	1.228
Race	0.322	1.230	0.817	1.853
HSCT	0.000	0.325	0.189	0.559
IMiDs vs IMiDs+PPAR agonists	0.029	0.559	0.332	0.941
